# Genome Sequences of Three Ross River Virus Isolates Obtained from the Australian Defence Force

**DOI:** 10.1128/MRA.00064-19

**Published:** 2019-04-04

**Authors:** Wenjun Liu, Joanne Kizu, Luke Le Grand, Ian Mitchell, Penelope J. Gauci, Aneta J. Gubala

**Affiliations:** a Australian Defence Force Malaria and Infectious Disease Institute, Enoggera, Queensland, Australia; b Land Division, Australian Defence Science and Technology Group, Fishermans Bend, Victoria, Australia; University of Southern California

## Abstract

The complete genome sequences of three Ross River virus (RRV) isolates from infected Australian Defence Force (ADF) personnel and from mosquitoes collected in ADF training areas were determined. Phylogenetic analysis in comparison with all available complete RRV nucleotide sequences from GenBank split these three RRV isolates into two distinct sublineages.

## ANNOUNCEMENT

Epidemic polyarthritis (EPA) caused by Ross River virus (RRV) infection is the most common notifiable arboviral disease in Australia, with approximately 5,500 cases reported annually (1). RRV is a positive-sense single-stranded enveloped RNA virus in the Alphavirus genus of the *Togaviridae* family (2, 3). There is no specific antiviral treatment and no commercial RRV vaccine available (4, 5). There are currently three recognized genotypes of the virus (6, 7).

Full genome sequences allow for intensive molecular evolutionary studies on vaccine development and disease control measures. There were only nine complete RRV genome sequences available in GenBank prior to our study, with the majority isolated more than 15 years ago from either mosquitoes or birds.

We present three RRV genome sequences collected from the Australian Defence Force (ADF). Two RRV strains (MIDI13.2016 and MIDI32.2017) were isolated from serum collected from ADF personnel presenting with EPA. The third strain (MIDI86.2018) was isolated from a homogenized pool of Verrallina funerea mosquitoes captured in a separate ADF training area in Queensland, Australia. All strains were isolated using C6-36 cells, an Aedes albopictus cell line (8). Total viral RNA was extracted from passage 1 of a 140-µl tissue culture supernatant using an RNeasy minikit (Qiagen), prior to being converted to cDNA using the REPLI-g whole-transcriptome amplification single-cell kit (Qiagen). The cDNA library was prepared using the Nextera XT kit and sequenced on a MiSeq instrument using a Reagent Micro kit version 2 (300 cycles; Illumina), according to the standard protocol, to produce approximately 1 million reads (2 × 150 nucleotides [nt]) for each sample. The sequence data were assembled by mapping the reads to a reference genome, that of RRV strain QML1 (GenBank accession number GQ433354), with the open reading frames and annotations determined by mapping each consensus sequence to QML1 with Geneious R11 (version 11.1.2), using default parameters at a low sensitivity setting. The complete RRV sequences were aligned using ClustalW, and a maximum clade credibility tree was generated using the Geneious (version 11.1.2) Bayesian phylogenetic method with an HKY85 substitution model, a gamma rate variation, and a chain length of 10^6^ with a burn-in length of 10^4^.

MIDI13.2016, MIDI32.2017, and MIDI86.2018 have a genome length of 11,892 nt, 11,894 nt, and 11,894 nt [without 3′poly(A)], G+C contents of 51.3%, 51.2%, and 51.2%, and an average sequence coverage of 11,491, 9,019, and 8,913 reads/nt, respectively. Phylogenetic analysis using Geneious (version 11.1.2) of all available strains of RRV in GenBank supports the assertion of Jones et al. that these three recent RRV isolates are highly conserved compared to the prototype strain T48 isolated in 1959, with maximum divergences of 3.9% and 1.8% for pairwise comparison of nucleotides and amino acids, respectively (7). The 99.5% nucleotide sequence identity between MIDI32.2017 and MIDI86.2018 isolates over a 2-year period from two different ADF training sites separated by more than 500 km indicates that genetic drift of RRV is quite restricted, suggesting that mutation/deviation from the prototypic sequence might have a dramatic impact on viral fitness and disease transmission.

**FIG 1:**
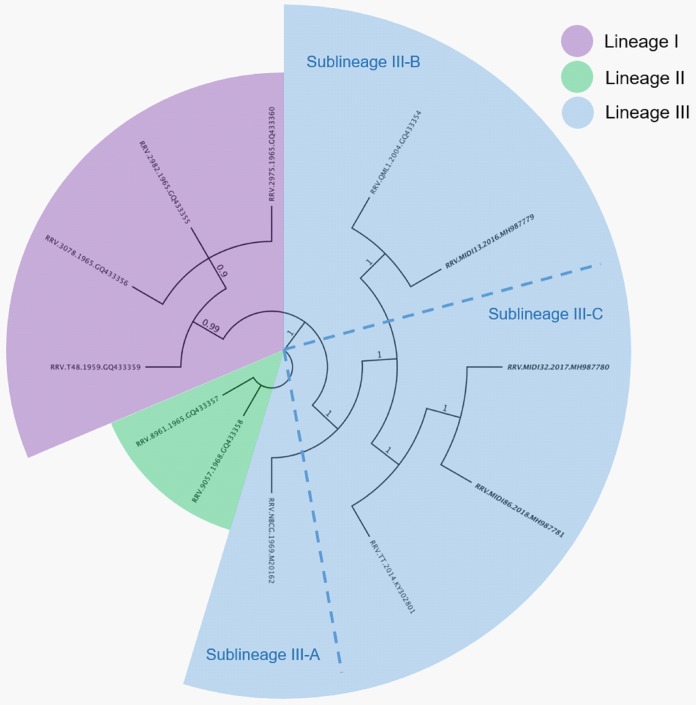
The maximum clade credibility tree based on all 12 complete RRV sequences in GenBank classified the ADF RRV isolates (seen in bold) into two distinct clades. Numbers at nodes indicate posterior probabilities. The strain nomenclature is formatted as follows: RRV.name of strain.year of isolation.GenBank accession number.

All three strains contain the repeated 12-amino acid (aa) sequence element in the *nsP3* gene, a characteristic of post-1979 RRV strains (9). Noticeably, there are 2 nt deletions in the subgenomic promoter region of MIDI13.2016 that are not seen in MIDI32.2017 and MIDI86.2018, warranting further investigation. This was confirmed by Sanger sequencing of a reverse transcription-PCR (RT-PCR) amplicon of the promoter subgenomic region. Phylogenetic analysis of all available whole-genome sequences in GenBank clustered MIDI32.2017 and MIDI86.2018 into a sublineage close to a 2014 isolate from a symptomatic blood donor (10) and MIDI13.2016 into a clade close to the 2004 human isolate QML1 (7) (Fig. 1).

### Data availability.

Raw next-generation sequencing (NGS) reads were deposited in the Sequence Read Archive under accession numbers SRX5370411, SRX5370412, and SRX5370413 and BioProject number PRJNA522026. The RRV genome sequences in this announcement are publicly available in GenBank under accession numbers MH987779, MH987780, and MH987781.
